# A MALDI-TOF-based Method for Studying the Transport of BBB Shuttles—Enhancing Sensitivity and Versatility of Cell-Based *In Vitro* Transport Models

**DOI:** 10.1038/s41598-019-40973-0

**Published:** 2019-03-19

**Authors:** Pol Arranz-Gibert, Bernat Guixer, Roger Prades, Sonia Ciudad, Ernest Giralt, Meritxell Teixidó

**Affiliations:** 1 Institute for Research in Biomedicine (IRB Barcelona), Barcelona Institute of Science and Technology (BIST), Baldiri Reixac 10, Barcelona, E-08028 Spain; 2 Department of Inorganic and Organic Chemistry, University of Barcelona, Martí i Franquès 1-11, Barcelona, E-08028 Spain

## Abstract

In recent decades, peptide blood-brain barrier shuttles have emerged as a promising solution for brain drugs that are not able to enter this organ. The research and development of these compounds involve the use of *in vitro* cell-based models of the BBB. Nevertheless, peptide transport quantification implies the use of large amounts of peptide (upper micromolar range for RP-HPLC-PDA) or of derivatives (*e.g*. fluorophore or quantum-dot attachment, radiolabeling) in the donor compartment in order to enhance the detection of these molecules in the acceptor well, although their structure is highly modified. Therefore, these methodologies either hamper the use of low peptide concentrations, thus hindering mechanistic studies, or do not allow the use of the unmodified peptide. Here we successfully applied a MALDI-TOF MS methodology for transport quantification in an *in vitro* BBB cell-based model. A light version of the acetylated peptide was evaluated, and the transport was subsequently quantified using a heavy internal standard (isotopically acetylated). We propose that this MALDI-TOF MS approach could also be applied to study the transport across other biological barriers using the appropriate *in vitro* transport models (*e.g*. Caco-2, PAMPA).

## Introduction

Peptide blood-brain barrier (BBB) shuttles^1^ are gaining relevance in drug delivery as they allow the transport of therapeutic agents into the central nervous system (CNS) at a lower economic cost than other BBB shuttle molecules such as monoclonal antibodies (mAbs)^2^. Additionally, peptide shuttles can be engineered to cross other endothelium barriers such those of the intestine^3^ or skin^4^. Furthermore, peptides have the advantages of small molecules from the production point of view, while simultaneously displaying both high specificity and affinity for a specific type of cell-receptor. These peptides allow transcytosis either through the BBB^5^ or another biological barrier, or high permeability with certain specificity through the lipid bilayer of the plasma membrane of endothelial cells^1^.

Several strategies can be used to discover new BBB shuttles. These include *in vitro* or *in vivo* phage design by means of computational tools, and the screening of natural sources or chemical libraries in *in vitro* BBB cell-based models (or non-cellular, *e.g*. PAMPA)^7–9^.

*In vitro* transport models, either cell-based or non-cellular, are key tools for research into active and passive transport, respectively^10–13^. The use of such models usually involves peptide transport quantification by reversed-phase high-performance liquid chromatography (RP-HPLC) with photodiode array (PDA) detection and identification by mass spectrometry (MS)^5^. Both cell-based and PAMPA models use a transwell system with donor and acceptor compartments. At the end of the assay, concentrations of the assayed molecule in the acceptor well are usually very low (one or two orders of magnitude lower compared to the donor well). Thus, RP-HPLC coupled to PDA often requires large amounts of peptide (upper micromolar range) in the donor compartment, thus implying the evaluation of these compounds at high concentrations and/or over long periods. In contrast, LC-MS/MS analysis—like selected reaction monitoring (SRM) methods—provide a high sensitivity and specificity towards the analyte^14,15^. Nevertheless, they usually require of sophisticated mass spectrometers, which increase the cost per analyzed sample.

Other methods may enhance detection sensitivity but lead to unsuitable modifications of the structure of the peptide. Attaching a fluorophore to the peptide—usually at the *N*-terminus—can increase detection up to the nanomolar range^16^. Nevertheless, the fluorophore can enhance peptide permeation through other mechanisms, as some have been described to be internalized by cells^17^. Similar issues are encountered with a stable isotope dilution methodology for cell-penetrating peptide (CPP) quantification in *in vitro* assays by MS^18,19^: the tag contains a biotin moiety that can be internalized by other mechanisms^20^. Although quantum dots could be used as fluorescent probes, several related issues such as size, coating, and high cost make them unsuitable for this purpose^21^. The use of radiolabeled peptides/proteins has also been described^22^ and although they allow an improvement in quantification, specific facilities and trained operators are required. Such a method involves a cumbersome manipulation process and often the labeling of tyrosine residues^22^, thus requiring the presence of this amino acid in the sequence. Moreover, side-chain labeling of peptides may interfere with the peptide-receptor interaction. In addition, compounds must be assayed in buffer^23^ when RP-HPLC-PDA is used for quantification, since cell culture media cannot be injected into the HPLC system—not at least in quantities required for PDA detection. Furthermore, the media components lead to peak-overlapping, and thus quantification cannot be performed. Therefore, these experimental constraints are an obstacle to achieve more reliable transport data and for mechanistic studies. Such studies require peptide concentrations around the limit between micro- and nanomolar ranges or even below in order to ensure non-saturated or non-crowded transport in carrier-mediated or endocytic mechanisms (*e.g*. receptor-mediated and adsorptive-mediated transport), respectively^24,25^.

Here we envisaged a method that, in an early screening stage, allowed the following: (1) evaluation of a peptide shuttle as a single molecule with minimal modification; (2) simultaneous identification (integrity assurance) and transport quantification; (3) improvement in data quality compared to that provided by RP-HPLC-PDA; (4) use of low peptide concentrations—a crucial feature for mechanistic studies; and (5) increase in the versatility of these *in vitro* models. We then designed a method based on MALDI-TOF MS, since MS is characterized by a high sensitivity, and MALDI instruments are characterized by their robustness without the need for an LC system coupled in line, in contrast to ESI MS.

## Results

### Design of a Peptide Shuttle Library

**Figure 1:**
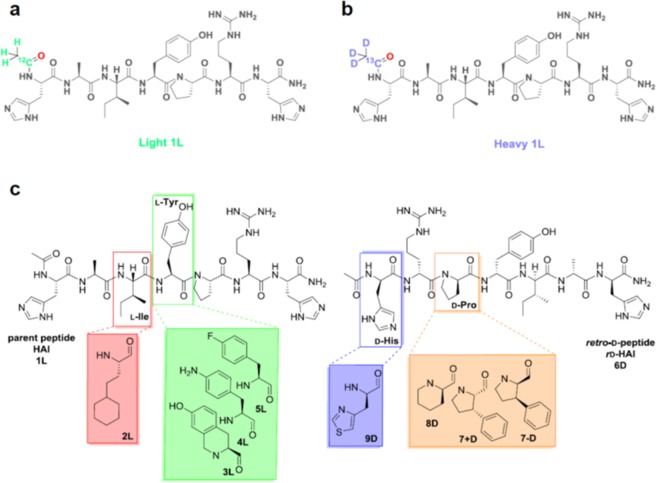
The H_2_N-HAIYPRH-CONH_2_ peptide BBB shuttle (1 L): (**a**) light and (**b**) heavy versions containing an isotopically labeled acetyl moiety; and (**c**) the library of analogs of the parent (HAI or 1 L) on the left, and the *retro*-D-version (*r*D-HAI or 6D) and its analogous modifications on the right.

HAI peptide, with the amino acid sequence HAIYPRH, was found by Lee *et al*. by phage display against the human transferrin receptor (TfR)^26^. TfR mediates the delivery of iron to the brain^27^ and is highly expressed in brain capillaries^28^. It is also expressed in choroid plexus epithelial cells and neurons^29^. The peptide was previously used in drug delivery^30^ and tumor-targeting^31^. In addition, we recently showed the peptide is an effective BBB shuttle *in vivo*, where gold nanoparticles were delivered into rat brains^32^. We initially designed a library of HAI-based BBB shuttles to study the effect of amino acid replacement on the capacity of HAI to cross the BBB: a set of HAI analogs containing single modifications in four of the seven available positions. Amino acid substitutions were chosen by side-chain analogy with the original residue in the parent peptide (Fig. 1c). Two analogy descriptors, bulkiness and bioisosterism^33,34^, were used to fine-tune the shuttle-receptor interaction. Hydrocarbon side-chains were substituted by either longer chains or analogs containing rings (saturated or aromatic). Heteroatoms were replaced by bioisosteres^33,34^ (OH in tyrosine by NH_2_ or F; imidazolyl in histidine by thiazolyl moiety), chemical substitutions with similar physicochemical properties which can exert similar but modulated biological effect. In addition, half the peptides were derivatives of *r*D-HAI^32^, the *retro-*enantio version (or *retro*-D) of HAI (Table 1). Therefore, in order to identify key residues involved in the ligand-receptor (peptide shuttle-TfR) interaction^35,36^, we studied the molecular features necessary for molecular recognition (*i.e*. the pharmacophore) of the peptide.

**Table 1:** List of the peptide analogs of HAI of and their residue substitutions. D-amino acids are shown in lowercase letters.

Peptide	Sequence	Substitution (“x”)
1 L (HAI)	Ac-HAIYPRH-NH_2_	—
2 L	Ac-HAxYPRH-NH_2_	homocyclohexyl-L-alanine
3 L	Ac-HAIxPRH-NH_2_	7-hydroxy-(*S*)-1.2.3.4-tetrahydroisoquinoline-3-carboxylic acid
4 L	Ac-HAIxPRH-NH_2_	4-amino-L-phenylalanine
5 L	Ac-HAIxPRH-NH_2_	4-fluoro-L-phenylalanine
6D (*r*D-HAI)	Ac-hrpyiah-NH_2_	—
7 + D	Ac-hrxyiah-NH_2_	(2*S*,3*S*)-3-phenylpirrolidine-2-carboxylic acid
7 − D	Ac-hrxyiah-NH_2_	(2*R*,3*R*)-3-phenylpirrolidine-2-carboxylic acid
8D	Ac-hrxyiah-NH_2_	D-pipecolic acid
9D	Ac-xrpyiah-NH_2_	4-thiazoyl-D-alanine

### Design of a MALDI-TOF MS Method for *in vitro* Transport Quantification

Precise peptide quantification by MS can be achieved only when using a standard with a similar mass and the same probability of desorption/ionization and detection. Thus, the ideal candidate for this purpose is the same peptide but isotopically labeled^37^. Here, in order to develop a new method suited for transport quantification by MS, instead of using isotopically labeled amino acids, we selected a small acetyl moiety with two isotopic versions—one light and one heavy. This molecule can be easily coupled at the *N*-terminus of peptides through a straightforward tagging protocol. The two acetyl isotopic versions are designed to avoid peak-overlap with the relevant isotopic peaks of the peptide shuttle (the second, third and fourth isotopic peaks for low molecular weight molecules–such as peptide shuttles) arising from the relative natural abundance of each atom isotope. To fulfill these requirements, we chose a heavy isotopically labeled acetyl moiety containing three deuterium atoms and one carbon-13 (Fig. 1b) and displaying +4 amu compared to the standard light version (Fig. 1a). Thus, the *N*-terminus of each peptide was differentially acetylated through the reaction with the respective symmetric anhydride (see Scheme S1, Table S1, Figs S1–S3). The presence of this acetyl moiety, which masks the NH_2_ terminus (positive) charge, resembles that of a cargo at the *N*-terminal of the shuttle.

### Transport Analysis of the Peptide Shuttle Library

**Figure 2:**
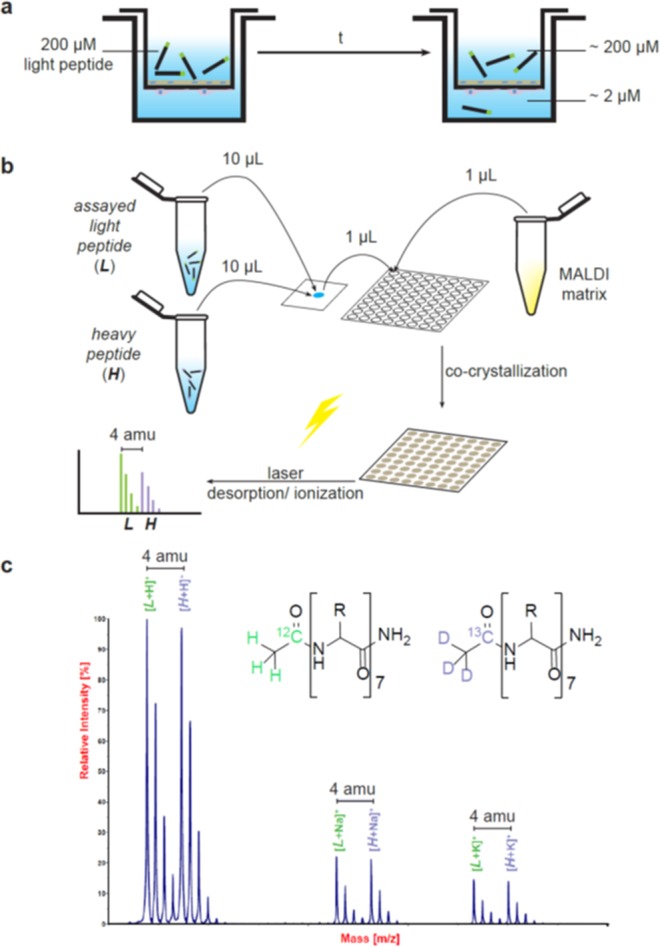
Scheme of the transport quantification method by MALDI-TOF MS. The *in vitro* BBB cell-based model (**a**) is performed in a transwell system where a membrane cultured with endothelial cells delimits two compartments (donor and acceptor). The donor compartment contains the light peptide before starting the experiment; at the end of the assay, a certain amount of peptide has been transported to the acceptor compartment (if 200 μM is assayed in the donor compartment, around 2 μM needs to be quantified in the acceptor compartment). Thus, (**b**) two aliquots of the heavy peptide are prepared, one at 200 μM and another at 2 μM; 10 μL of the assayed light peptide (from acceptor or donor compartments) and 10 μL of the heavy version, at similar concentrations, are mixed. Subsequently, 1 μL of this mixture and 1 μL of an appropriate MALDI matrix (*e.g*. ACH matrix solution) are placed on a MALDI plate. Finally, the spectra are acquired. A spectrum (**c**) obtained from a solution containing light and heavy versions of 9D peptide at 2 μM is shown. Light and heavy peptides are observed as the *m/z* of the peptides plus H^+^, Na^+^ or K^+^. In all cases, isotopic homolog peaks between light and heavy peptides display a 4-amu mass difference.

Peptides were evaluated through an *in vitro* bovine BBB cell-based model (Fig. 2a). Transport (*T*) and permeability (*P*_*app*_) for all peptides were quantified by RP-HPLC-PDA and by MALDI-TOF MS (Fig. 2). The former method determined transport by applying the ratio between the peak areas integrated in the chromatograms from the acceptor and donor wells, further corrected by the volumes injected and the volumes contained in each well (see Eq. 1). The latter entails the spiking of the heavy version of the peptide as internal standard into an aliquot of the light peptide (Fig. 2b). The MS intensity for light/heavy ratios for acceptor and donor wells is representative of the relative amount of peptide in each well when corrected by their volume (detailed in Eq. 2).

1 $$T=\,\frac{{Q}_{A}^{I}(t)}{{Q}_{D}^{I}({t}_{0})}\times \frac{{V}_{D}^{I}}{{V}_{A}^{I}}\times \frac{{V}_{A}^{W}}{{V}_{D}^{W}}\times 100\,( \% )$$

where ${Q}_{A}^{I}(t)$ and ${Q}_{D}^{I}({t}_{0})$ account for the integrated area in the HPLC chromatograms of acceptor (at time *t* = 2 h) and donor (at time *t*_0_ = 0) wells, respectively; ${V}_{D}^{I}$ and ${V}_{A}^{I}$ are the injected volumes from donor and acceptor wells, respectively; and ${V}_{A}^{W}$ and ${V}_{D}^{W}$ are the volumes in each acceptor and donor well, respectively.

2 $$T=\frac{{Q}_{A}^{Light}(t)/{Q}^{Heavy}}{{Q}_{D}^{Light}({t}_{0})/(R\,\times \,{Q}^{Heavy})}\times \frac{{V}_{A}^{W}}{{V}_{D}^{W}}\times 100\,({\rm{ \% }})$$

${Q}_{A}^{Light}(t)/{Q}^{Heavy}$ accounts for the relative amount of the light peptide in the acceptor well (at time *t*) compared with a prepared dilution of the heavy isomer (at 2 μM). ${Q}_{D}^{Light}({t}_{0})/(R\times {Q}^{Heavy})$ determines the relative amount of light peptide in the donor well (at time *t* = 0) compared with a prepared dilution of the heavy isomer (at 200 μM; *i.e*. in our case *R* = 100).

In general, transport (see Eq. 3) can be described by the total amount of peptide in the acceptor well at time *t*, ${Q}_{A}(t)$, divided by the initial amount in the donor well, ${Q}_{D}({t}_{0})$, while apparent permeability (*P*_*app*_), widely used in the literature, normalizes transport by the transwell area, volume in donor well and time (see Eq. 4). To simplify discussion, results are discussed in terms of transport normalized by the parent peptide (1 L).

3 $$T=\frac{{Q}_{A}(t)}{{Q}_{D}({t}_{0})}\times 100\,( \% )$$

4 $${P}_{app}=\frac{d{Q}_{A}(t)}{dt}\frac{1}{A}\frac{1}{{Q}_{D}({t}_{0})}{V}_{D}\approx \frac{{Q}_{A}(t)}{t}\frac{1}{A}\frac{1}{{Q}_{D}({t}_{0})}{V}_{D}=\frac{{V}_{D}}{t}\frac{1}{A}\frac{{Q}_{A}(t)}{{Q}_{D}({t}_{0})}=\frac{{V}_{D}}{t}\frac{1}{A}\frac{T}{100}(cm/s)$$

All the peptides were assayed at 200 μM to ensure maintenance above the RP-HPLC-PDA limit of quantification (LOQ) (assuming a value of 2% transport, around 1 μM of peptide needs to be quantified –when concentrations are corrected by donor/acceptor well ratio, *i.e*. by dividing additionally per 4). In spite of the high amount of peptide assayed, quantification by RP-HPLC-PDA was below the LOQ in some cases (Fig. S7a). In these cases, an approximate value was determined.

Initially, the data obtained by these two methods was compared by analyzing differences in transport within the same sample as mean ± standard deviation (SD) (Table S3, Fig. S6). Small discrepancies were observed but no significant differences were detected.

**Figure 3:**
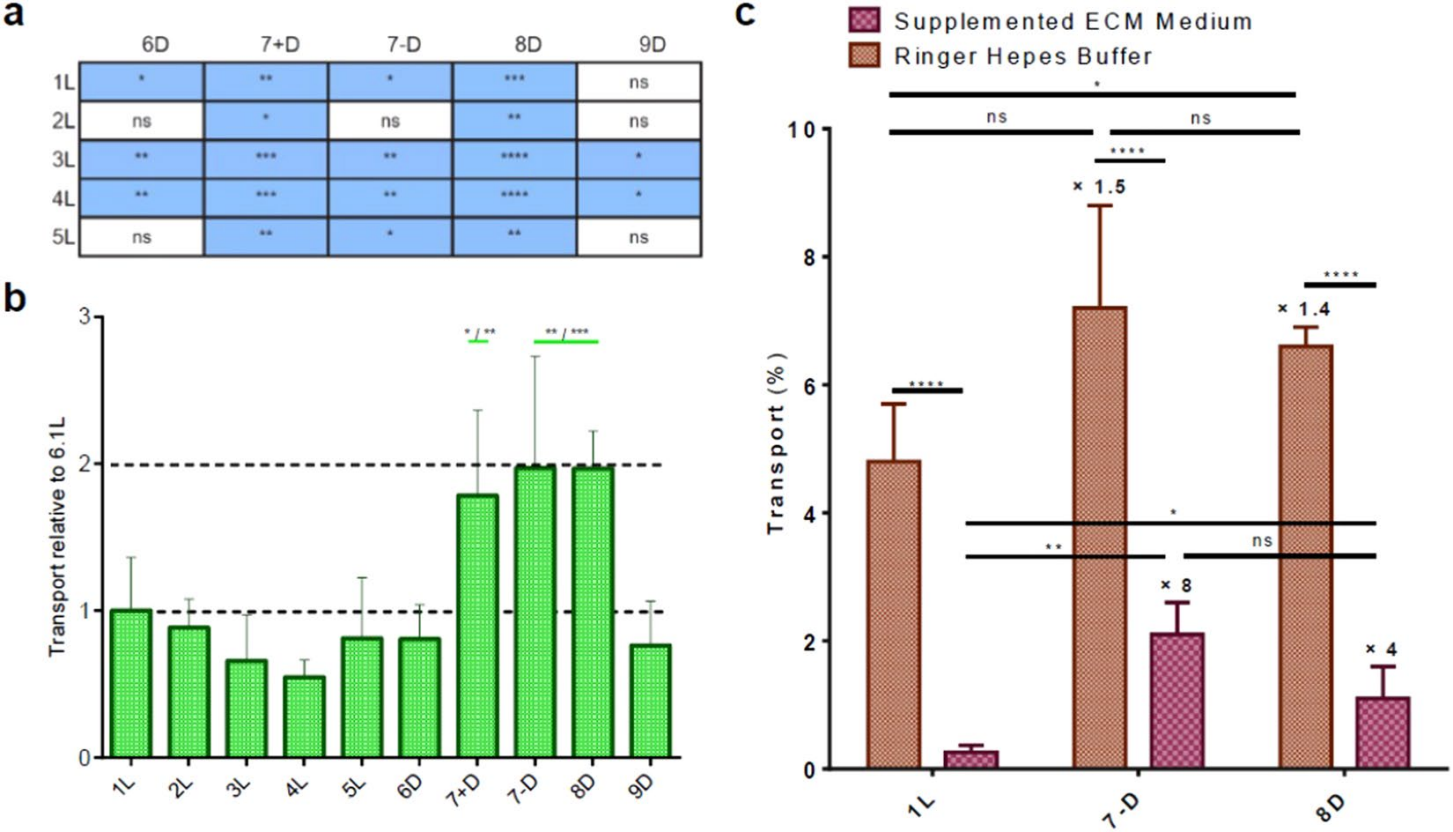
Peptide transport results from the *in vitro* bovine BBB cell-based model assay obtained through RP-HPLC-PDA (in blue) or MALDI-TOF MS (in green) quantification; Data are expressed as mean ± SD, n = 3. (**a**) RP-HPLC-PDA transport quantification significance between L- and D-peptides (additionally, peptide 9D showed significant differences (*) with 7 + D and 8 D); (**b**) MALDI-TOF MS transport quantification results and significance. Differences in group means were assessed using a linear model fitted for each measurement method. In each case, Wald tests derived from the linear models were used to perform pairwise comparisons between experimental conditions. A 5% level was chosen for significance of group. *In vitro* human BBB model: (**c**) transport of 1 L, 7 − D and 8D peptides when assayed with Ringer-HEPES buffer or supplemented ECM medium. Extremely significant differences (****) are observed when peptides are assayed in buffer or medium; within peptides assayed in buffer, significant differences (*) are only observed between 1 L and 8 D; within peptides assayed in medium, significant differences (* or **) are observed between 1 L both *retro*-D-analogs (7 − D and 8 D). Significance: ns ≡ not significant (p ≥ 0.05), *significant (0.01 ≤ p < 0.05), **very significant (0.001 ≤ p < 0.01), ***extremely significant (0.0001 ≤ p < 0.001), ****extremely significant (p < 0.0001). Data analysis was done using the T test (non-parametric).

Nevertheless, significance within the same method differed considerably (Fig. 3a,b). RP-HPLC quantification displayed the main significant differences in transport between D-peptides (6D, 7 + D, 7 − D, 8D and 9D) and L-peptides (1 L, 2 L, 3 L, 4 L and 5 L). On the basis of this observation, one could infer that the D-peptides showed higher transport, probably owing to the labile structure of L-peptides in a biological environment (*i.e*. in an *in vitro* BBB cell-based model –the BBB is known to also be an enzymatic barrier)^13,38,39^ and the greater stability of D-peptides conferred by the inverse configuration. Only peptide 9D showed significant differences with other D-peptides (7 + D and 8D) (Fig. 3a, Table S3, Fig. S6).

On the other hand, MALDI-TOF MS quantification evidenced the relevance of proline substitution by any of the three cyclic amino acid analogs (in peptides 7 + D, 7 − D and 8D) as transport enhancers (Fig. 3b). Significant differences in transport were observed only for peptides 7 + D, 7 − D and 8 D when compared with the rest. These peptides showed a two-fold increase in transport compared to the parent peptide (peptide transport normalized by the parent peptide (1 L) is shown in Fig. 3b). Thus, the proline residue in *r*D-HAI seems to be a key pharmacophoric site^35,40^ that leads to an improvement in transport when substituted by a bulkier residue.

Finally, to further study their transport ability and test the versatility of our method for quantification, we selected the peptides that showed the greatest transport capacity, namely 7 − D and 8D (7 + D was discarded as it recorded the same transport as 7 − D, but the residue replaced is an L- instead of D-amino acid). Interestingly, we have previously reported the use of the non-natural amino acids 7 − D and 7 + D for passive diffusion transport purposes. In that case, the amino acid in question (PhPro, *i.e*. phenylproline) was used as a building block for the tetrapeptide PhPro_4_, which showed excellent passive diffusion transport^1^. Passive diffusion was ruled out as the mechanism responsible for transport when the peptides (1 L, 6D, 7 − D and 8D) were assayed by PAMPA, an *in vitro* physico-chemical model where the transport by passive diffusion of compounds is evaluated through a membrane containing the lipid composition of interest (in our case, a porcine brain polar lipid extract (BPLE) to mimic the BBB lipid barrier). All of the peptides showed permeability values below 0.4 × 10^−6^ cm/s (Fig. S5 and Table S2). Note that when these values fall below 2.0 × 10^−6^ cm/s compounds are considered to be poorly transported by passive diffusion^41^.

We tested the protease resistance (in human serum) of these two *all*-D-peptides containing a non-natural moiety (7 − D and 8D). Both showed high resistance to protease degradation, as previously observed for the *retro*-D-version of HAI, *r*D-HAI (Fig. S4). With these optimal candidates, we moved on to a more sophisticated *in vitro* human model of the BBB, made using brain-like endothelial cells, generated from human cord blood-derived hematopoietic stem cells co-cultured with bovine pericytes. The aforementioned peptides were assayed as in the other BBB model using either Ringer-HEPES buffer (the one commonly used in these assays) or supplemented ECM medium. The use of medium in this assay allows (1) the extension of the incubation time and (2) the use of more similar conditions to those found physiologically. In this assay, the transport of the analogs 7 − D and 8D was around 1.5 times higher than that observed for the parent peptide 1 L (HAI) when using buffer. However, when 7 − D and 8D were assayed in medium, they showed eight- and four-fold times the transport of the parent peptide (Fig. 3c).

### Performance and Broader Applicability of the MALDI-TOF MS Method for Transport Quantification

To further analyze the results and compare the two methods (MALDI-TOF MS and RP-HPLC-PDA), the transport of each replica was compared individually (Fig. S6). Consistent with the previous results, although small differences were revealed, the relative discrepancy between the two methods (RP-HPLC-PDA and MALDI-TOF MS) remained around 20% (Table S3). These discrepancies are most likely due to RP-HPLC-PDA quantification errors since some of the acceptor wells were below the LOQ of this technique (Figs S7 and S9), although we previously optimized the wavelength selected (Fig. S8, Table S4).

**Figure 4:**
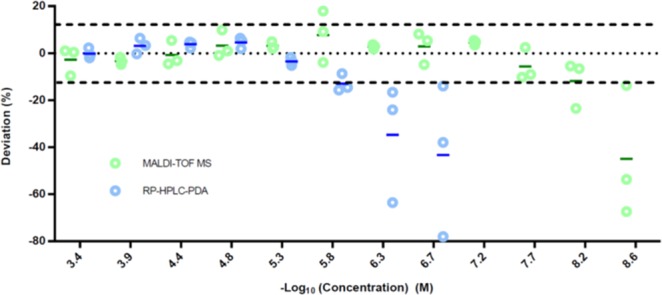
Determination of the limit of quantification of MALDI-TOF MS and RP-HPLC-PDA using the peptides 1 L, 6D and 9D.

To determine the LOQ of our MALDI-TOF MS approach, we analyzed consecutive dilutions (from 200 μM to 1 nM) of samples of 1 L (HAI), 6D (*r*D-HAI) and 9 D, which contained light and heavy versions in similar concentrations (the latter double the concentration of the former). Each dilution was analyzed by comparing the light/heavy ratio with the initial quantification before dilution. The LOQ remained around 3.4 nM in both cases when an average error of 8% was assumed (Fig. 4). This value is much lower than the discrepancy between replicates in this type of assay (Table S3, Fig. S6). Thus, MALDI-TOF MS allowed an increase in sensitivity of more than 3 orders of magnitude compared to RP-HPLC-PDA. Accordingly, all transport quantifications by MALDI-TOF MS fell within the LOQ.

## Discussion

HAI peptide has been widely applied for TfR targeting in cancer, where this receptor is overexpressed. We previously expanded its applicability as BBB shuttle by initially studying its mechanism of endocytosis/transcytosis and evaluating its capacity as a shuttle to transport nanoparticles *in vivo* (in rat), where the peptide showed promise.

In order to improve its transport features, including protease stability and transport activity, we designed a library of ten analogs, half based on the parent peptide and half on the *retro*-D-version. At the same time, we wanted to improve the current methods for transport quantification using cell-based *in vitro* models of the BBB. A MALDI-TOF MS method based on isotopic labeling using an acetyl moiety was devised. RP-HPLC coupled to PDA was used as standard method to compare the performance and applicability of the novel method. Both methods were chosen for their simplicity in quantification; however, only MALDI-TOF MS allows (1) identification during quantification, (2) analysis of complex samples (*e.g*. those containing cell culture medium) without a pretreatment (required for LC systems) and (3) an increase in sensitivity.

Discrepancies found between the transport quantification using the two methods were most likely due to RP-HPLC-PDA quantification errors since some of the acceptor wells were below the LOQ of this technique. Indeed, these data suggested that MALDI-TOF MS is a more suitable tool for transport quantification than RP-HPLC-PDA, since we were evaluating an already described hit (HAI) and its analogs at relatively high concentration (200 μM), all of them containing 3 aromatic rings (two histidine and one tyrosine residues, or analogs) and thus displaying a relatively high UV absorption.

In our MALDI-TOF MS method, we propose that two procedures can be followed in order to ensure that the concentration of the assayed peptide is above the LOQ. The most stringent one requires the determination of the LOQ for each peptide. The second one considers that similar molecules (our library of peptide analogs) have similar ionization properties. In our case, MALDI-TOF MS allowed an increase in sensitivity of more than 3 orders of magnitude compared to RP-HPLC-PDA. Accordingly, all transport quantifications by MALDI-TOF MS fell within the LOQ.

Analogs 7 + D, 7 − D and 8 D showed a two-fold increase in transport compared to the parent peptide (Fig. 3b), when the library was assayed in the bovine *in vitro* model of the BBB and transport was analyzed by MALDI-TOF MS. The three peptides have a single substitution in the proline residue of *r*D-HAI by a bulkier amino acid. Although 7 − D and 8D appeared to show similar transport performance using the *in vitro* bovine BBB model, a more realistic analysis of their shuttle capacity (using a human model) revealed that 7 − D was twice as good as 8D, in spite of the latter exceeding the transport capacity of the parent peptide (Fig. 3c). They showed an increase in transport of eight- and four-fold compared to the parent peptide, respectively.

The novel MALDI-TOF MS-based method for transport quantification of peptide shuttles has several advantages: (1) the acetyl moiety is the smallest tag possible to incorporate into the peptide through a straightforward methodology; (2) it does not influence the uptake of the peptide; (3) it mimics the charge state of the *N*-terminus when a cargo is attached, as previously described; (4) it is not cleaved from the peptide; and finally, (5) it is easily incorporated into any peptide without any requirement of a specific residue on the peptide sequence. In addition, it allows (6) the simultaneous identification (integrity assurance) and transport quantification; (7) improvement in data quality compared to that provided by RP-HPLC-PDA; (8) use of low peptide concentrations—a crucial feature for mechanistic studies; and (9) increase in the versatility of these *in vitro* models. Moreover, this methodology can be used to quantify peptides for other purposes: in other cell-based transport models (*e.g*. the Caco-2 intestinal barrier model) or to determine their physicochemical properties (*e.g*. the determination of the Log P may be tedious if this parameter is higher than 3 due to limitations in the sensitivity of methods available).

## Conclusion

We have shown how *in vitro* models can facilitate the identification and development of BBB shuttle candidates. Moreover, we demonstrate that using these models to analyze the transport of a chemically-designed library of analogs can serve to improve the performance of the shuttles identified. To explore the relevance of each residue, we devised a library of ten peptides half derived from the parent peptide and half from the *retro*-D-version. Such derivatives had a single residue substitution by a non-natural analog. Thus, to evaluate these compounds, we envisaged a method for precise quantification while enhancing the versatility of *in vitro* models of the BBB by means of MALDI-TOF MS and isotopic labeling. Using a subtle modification (*i.e. N*-terminal acetylation), the novel method allowed an increase in sensitivity by three orders of magnitude compared with a standard method (RP-HPLC-PDA), while revealing a two-fold increase in transport in three analogs containing the same residue permutation (Pro). Furthermore, it enabled the quantification of samples containing complex cell media, thereby providing new data on the biostability and bioactivity of these so-called BBB shuttles without any kind of sample processing.

## Methods

### Materials and Methods

Protected amino acids and resins were supplied by Luxembourg Industries (Tel-Aviv, Israel), Neosystem (Strasbourg, France), Calbiochem-Novabiochem AG (Laüfelfingen, Switzerland), PolyPeptide Laboratories (Torrance, CA, USA), Bachem AG (Bubendorf, Switzerland), and Iris Biotech (Marktredwitz, Germany). COMU and Oxyma Pure were purchased from Calbiochem-Novabiochem AG. Acetic acid-1-^13^C,d_4_ was obtained from Aldrich (Milwaukee, WI, USA). DIEA and ninhydrin were from Fluka Chemika (Buchs, Switzerland). Solvents for peptide synthesis and RP-HPLC were supplied by Scharlau or SDS (Barcelona, Spain). Trifluoroacetic acid was purchased from KaliChemie (Bad Wimpfen, Germany). The other chemicals used were from Aldrich (St. Louis, MO, USA) and were of the highest purity commercially available. PAMPA plates and PAMPA system solution were from pION (Woburn, MA, USA). Porcine brain polar lipid extract was supplied by Avantis Polar Lipids (Alabaster, AL, USA). NMR experiments were carried out on a Bruker Avance III 600 MHz spectrometer equipped with a TCI cryoprobe. Mass spectra were recorded on an Applied Biosystems 4700 MALDI-TOF spectrometer (PE Applied Biosystems, Foster City, CA, USA), using an ACH matrix. High-resolution mass spectra were recorded on a Synapt HDMS (Waters, Manchester, UK) and on a LTQ-FT Ultra (Thermo Scientific, Waltham, MA, USA). RP-HPLC chromatograms were recorded on a Waters model Alliance 2695 with photodiode array (PDA) detector 996 from Waters (Waters, Milford, CT, USA) using a SunFire C_18_ column (150 × 4.6 mm × 5 µm, 100 Å, Waters); solvents: H_2_O (0.045% TFA) and CH_3_CN (0.036% TFA); flow rate of 1 mL/min; and software Millenium version 4.0. HPLC-MS [Waters model Alliance 2796, quaternary pump, Waters 2487 with UV/Vis dual absorbance detector, ESI-MS model Micromass ZQ and Masslynx version 4.0 software (Waters)] was done using a SunFire C_18_ column (150 × 3.9 mm × 5 µm, 300 Å, Waters); solvents: H_2_O (0.1% formic acid) and CH_3_CN (0.07% formic acid); and flow rate of 1 mL/min. The products were purified in a 2545 binary gradient module, a 2767 manager collector, and a 2998 photodiode array (PDA) detector (Waters), with Masslynx version 4.1 (Waters), and a SunFire C_18_ column (150 × 10 mm × 5 µm, 100 Å, Waters); solvents: H_2_O (0.1% TFA) and CH_3_CN (0.1% TFA); and flow rate of 3 mL/min. Graphics were performed using GraphPad Prism version 6.01 for Windows (GraphPad Software, La Jolla, CA, USA).

### General Protocol for SPPS

Peptides were synthesized using Fmoc/*t*Bu SPPS standard protocols. In all cases, Fmoc-Rink-Amide AM resin^42,43^ (100 μmols) was used. Standard solid-phase peptide elongation and other solid-phase manipulations were done manually in polypropylene syringes, each fitted with a polyethylene porous disk at the bottom. Solvents and soluble reagents were removed by suction. Between couplings and deprotections, the resin was washed with DMF (5 × 1 min), DCM (5 × 1 min), and DMF (5 × 1 min), using 5 mL of solvent/g of resin each time. During couplings, the syringe was left under automatic stirring. Intermittent manual stirring was applied during deprotections. A diagram of the synthetic strategy for HAI analogs is shown in Scheme S1. After each reaction, the Kaiser test^44^ was used to identify primary amines on the *N*-terminus of the elongating peptide on the solid support. The chloranil test^45^ was used to identify secondary amines. Resin was conditioned by washing with DCM (5 × 1 min) and DMF (5 × 1 min), followed by 20% piperidine in DMF (1 × 1 min, 2 × 10 min) to remove the Fmoc group. Finally, the resin was washed with DMF (5 × 1 min). Fmoc group was removed by treating the resin with 20% piperidine in DMF (3–4 mL/g of resin; 1 × 1 min, 2 × 10 min). For secondary amine Fmoc deprotection, resin was additionally treated with DBU, toluene, and piperidine in DMF (5:5:20:70, v/v) (1 × 10 min). *N*^α^-Fmoc-protected amino acid (4 eq.), COMU^46^ (4 eq.), and Oxyma Pure^47^ (4 eq.) were added sequentially to the resin in DMF (minimal volume to allow the complete dissolution of the reagents), followed by DIEA (8 eq.). The mixture was allowed to react under stirring in an orbital shaker for 1 h. Afterwards, the solvent was removed by filtration, and the resin was washed with DMF (5 × 1 min) and DCM (5 × 1 min). The extent of coupling was checked by the appropriate colorimetric test. When required, a recoupling step was performed using the same previous conditions but for longer (2 h).

### *N*-Terminal Capping

The anhydride of the acetic acid (4 eq.) was prepared by mixing acetic acid (8 eq.) with DIPCDI (2:1) and adding 1 mL of DCM. Two minutes of agitation was applied. Afterwards, the solvent was added to the SPPS syringe, and the precipitate (*N*,*N′*-diisopropylurea) was discarded. DCM was added until all the resin was covered, and DIEA (4 eq.) was then added. After 30 min with stirring in the orbital shaker, the solvent was removed by filtration, and the resin was washed with DCM (5 × 1 min), DMF (5 × 1 min) and DCM (5 × 1 min). The extent of coupling was checked by the appropriate colorimetric test.

### Peptide Cleavage and Work-Up

In order to cleave the peptides from the resin, the cleavage cocktail (TFA/TIS/H_2_O, 95:2.5:2.5, v/v; 8 mL) was added to each syringe. Intermittent manually agitation was applied for 30 min. The solvent was then collected in a plastic tube, and a new cleavage cocktail was added (8 mL) to the syringe. After 30 min, the solvent was collected in the same plastic tube and evaporated by a N_2_ flow. After evaporation of the cleavage cocktail, 20 mL of methyl *tert*-butyl ether (MTBE) was added to the plastic tube containing the dry residue. This tube was centrifuged at 2,000 × *g* for 10 min. The solvent was then discarded by decantation. This process was repeated two more times. After the last washing with MTBE, the final residue was dissolved in H_2_O/CH_3_CN (1:1) and then lyophilized.

### Peptide Purification and Characterization

Peptides were purified by RP-HPLC using a SunFire C_18_ column. Compound identity was confirmed using MALDI-TOF MS and HRMS. Peptide purity was checked by RP-HPLC using a SunFire C_18_ column. Additionally, ^1^H-NMR (600 MHz) experiments confirmed the identity and purity of the set of pure HAI analogs, as well the proper isotopic labeling between pairs of peptides (either acetylated with acetic acid or with acetic acid-1-^13^C,d_4_). Samples were prepared by dissolving peptides in H_2_O/D_2_O 80:20, v/v. Suppression of the water signal was achieved by WATERGATE W5^48^. All experiments were performed at 298 K.

### *In vitro* Bovine BBB Cell-Based Model Assay

This assay was an adapted model^5^ of the method previously published by Gaillard and de Boer^49^. Before performing the assay, TEER was measured in all transwells (TEER > 100 Ω·cm^2^). Peptides were prepared at a concentration of 200 μM in Ringer-HEPES buffer containing 20 μM Lucifer yellow (LY) lithium salt (Sigma-Aldrich) as control (P_app_ < 17·10^−6^ cm/s). The apical compartment was filled with 200 μL of the solution containing the peptide, and 800 µL of Ringer-HEPES was poured into the basal well. Three replicates of each peptide were assayed. The plate was left for 2 h in the incubator at 37 °C. Finally, the samples were collected and analyzed or frozen until analysis. LY fluorescence was measured in a 96-well plate with a Fluoroskan Ascent Microplate Fluorometer (Thermo Fisher Scientific). Two parameters were determined, namely transport (*T* (%)) and apparent permeability (*P*_*app*_(*cm*/*s*)). RP-HPLC-PDA (220 nm) and MALDI-TOF MS were used to determine the amounts of peptide in each sample.

### RP-HPLC-PDA Limit of Quantification

Dilution series for three peptides 1 L (HAI), 6D (*retro*-D-HAI) and 9D, ranging from 200 µM to 1.1 nM, were analyzed by RP-HPLC-PDA. An initial solution at 200 µM was consecutively diluted 1/3 by mixing 100 µL in 200 µL of H_2_O, up to a total of eleven dilutions. Finally, a specific volume of the sample, ranging from 5 to 100 µL, was injected to the HPLC system and then further analyzed to determine the limit of quantification. First, the total absorption corrected by the injected volume and dilution was determined (${R}_{i}=\frac{A\times d}{V}$). *A*, *V* and *d* are the total absorption area, the absorption area, the injected volume and the fold-dilution, respectively. Then, the R_LOQ_ was determined (${R}_{LOQ}=\frac{{R}_{i}-\bar{R}}{\bar{R}}=\frac{{R}_{i}}{\bar{R}}-1$). $\bar{R}$ is the mean (*i.e*. $\bar{R}\equiv \sum _{i}^{n}\frac{{R}_{i}}{n}$; last five dilutions discarded). The deviation (%) is calculated as ${R}_{LOQ}\times 100$.

### MALDI-TOF MS Transport Quantification

Transport was calculated using Eq. 2. Hence, MS spectra intensities of light (assayed) and heavy peptides from acceptor and donor wells are representative of the relative amount of peptide in each well when corrected by their volume. Peak-picking and determination of peak area ratios was performed using Data Explorer version 4.5 (Applied Biosystems).

### MALDI-TOF MS Limit of Quantification

Dilution series for three peptides 1 L (HAI), 6D (*retro*-D-HAI) and 9D, ranging from 200 µM to 1.1 nM, were analyzed by MALDI-TOF MS (on an Applied Biosystems 4800 Plus MALDI-TOF spectrometer, using an ACH-based matrix). The initial dilution was prepared by mixing 20 µL of two solutions containing 400 or 800 µM of the light or heavy versions of the peptide, respectively. Consecutive dilutions 1/3 were then prepared by mixing 10 µL in 20 µL of H_2_O. Finally, 1 µL of the sample and 1 µL of the ACH matrix were placed in a MALDI plate. The spectra were acquired and further analyzed to determine the limit of quantification. First, the experimental light/heavy ratio was determined for all the dilutions. The R_LOQ_ was then determined for all of them (${R}_{LOQ}=\frac{{R}_{i}-\bar{R}}{\bar{R}}=\frac{{R}_{i}}{\bar{R}}-1$). ${R}_{i}$ and $\bar{R}$ are the light/heavy ratio of each sample and the mean (*i.e*. $\bar{R}\equiv \sum _{i}^{n}\frac{{R}_{i}}{n}\,$, discarding the last dilution value), respectively. The deviation (%) is calculated as ${R}_{LOQ}\times 100$.

### *In vitro* Human BBB Cell-Based Model Assay

This assay was performed using the model published by Prof. Cecchelli in^50^. Endothelial cells and pericytes were defrosted in gelatin-coated Petri dishes (Corning). Pericytes and endothelial cells were cultured in DMEM pH 6.8 or in supplemented endothelial cell growth medium (Sciencells), respectively. After 48 h, pericytes (50,000 cells/well) and endothelial cells (80,000 cells/well) were seeded in gelatin-coated 12-well plates or in Matrigel-coated 12-well Transwell inserts (Corning), respectively. Medium was changed every 2–3 days and assays were performed 7–8 days after seeding. Lucifer Yellow (50 µM) was used as internal control (*P*_*app*_ < 15·10^−6^ cm/s). LY fluorescence was measured in a 96-well plate with a Fluoroskan Ascent Microplate Fluorometer (Thermo Fisher Scientific). Compounds were dissolved in Ringer-HEPES at a concentration of 200 µM. Then, 500 µL of the compound and 1,500 µL of Ringer-HEPES alone were introduced in the apical or in the basolateral compartments, respectively. The plates were set on at 37 °C for 2 h. The solutions from both compartments were then recovered and quantified by HPLC and identified by MALDI-TOF.

### Peptide Stability in Human Serum

Peptides were dissolved in 1 mL of HBSS buffer/human serum (from human male AB plasma; Sigma-Aldrich) 10:90 (v/v) at a final concentration of 200 µM and incubated at 37 °C for 24 h. Aliquots of 50 µL were extracted at a range of incubation times and treated with 200 µL of cold CH_3_OH (4 °C) to precipitate serum proteins. After 30 min, samples were centrifuged at 13,000 g and 4 °C for 30 min. Supernatants were analyzed by RP-HPLC-PDA and MALDI-TOF MS, using similar procedures as for the quantification of the *in vitro* cell-based BBB model.

### Parallel Artificial Membrane Permeability Assay (PAMPA)

The PAMPA assay was used to exclude passive diffusion capacity across the BBB. The standard parameter that quantifies transport independently of time and concentrations is effective permeability, ${P}_{e}=\frac{-218.3}{t}Log(1-\frac{2{C}_{A}(t)}{{C}_{D}({t}_{0})})\times {10}^{-6}cm/s$; where *t* is the running time (4 h), *C*_*A*_ *(t)* is the concentration of the compound in the acceptor well at time *t*, and *C*_*D*_ *(t*_0_) is the compound concentration in the donor well before running the PAMPA assay (*t*_0_ = 0 h). Transport (%) values were obtained by dividing the amount of peptide in the acceptor well at time *t*, *C*_*A*_ *(t)*, by the amount in the donor well at time zero, *C*_*D*_ *(t*_0_), multiplied by 100. The protocol followed was the same as that previously used by Arranz-Gibert *et al*.^1^. Permeability is considered excellent with values >4.0 × 10^−6^) cm/s, uncertain between 2.0 × 10^−6^ and 4.0 × 10^−6^ cm/s, and poor with values below 2.0 × 10^−6^) cm/s^41^.

### Statistical Analysis

Differences in group means (in the *in vitro* BBB transport assay of the full set of HAI analogs–analyzed and quantified using RP-HPLC-PDA and MALDI-TOF MS) were assessed using a linear model fitted for each measurement method. In each case, Wald tests derived from the linear models were used to perform pairwise comparisons between experimental conditions. A 5% level was chosen for significance of group.

## Supplementary information

Supplementary information arranz et al

## Data Availability

All the relevant data supporting the findings are available from the corresponding author on reasonable request.
