# Supplementary material for: A MALDI-TOF-based Method for Studying the Transport of BBB Shuttles—Enhancing Sensitivity and Versatility of Cell-Based In Vitro Transport Models
Source: Sci Rep. 2019 Mar 19;9:4875. doi: 10.1038/s41598-019-40973-0 (PMC6424956; doi:10.1038/s41598-019-40973-0)
Supplement: Supplementary file 1 — Supplementary information arranz et al [file 41598_2019_40973_MOESM1_ESM.docx]

**SUPPORTING INFORMATION**

**A MALDI-TOF-based Method for Studying the Transport of BBB Shuttles—Enhancing Sensitivity and Versatility of Cell-Based *In Vitro* Transport Models**

Pol Arranz-Gibert,^a#^ Bernat Guixer,^a#^ Roger Prades,^a^ Sonia Ciudad,^a^ Ernest Giralt,^a,b*^ Meritxell Teixidó^a*^

Affiliations:

a) Institute for Research in Biomedicine (IRB Barcelona), Barcelona Institute of Science and Technology (BIST), Baldiri Reixac 10, Barcelona, E-08028, Spain.

b) Department of Inorganic and Organic Chemistry, University of Barcelona, Martí i Franquès 1-11, Barcelona, E-08028, Spain.

# These authors contributed equally.

**Corresponding authors (*):**

Dr. Meritxell Teixidó and Prof. Ernest Giralt, Institute for Research in Biomedicine (IRB Barcelona), Baldiri Reixac 10, Barcelona, E-08028, Spain. Tel.: +34 93 4037125, Fax: +34 93 4037126.

E-mail: ernest.giralt@irbbarcelona.org, meritxell.teixido@irbbarcelona.org

**Table of Contents**

Solid-Phase Peptide Synthesis S3

Characterization of the Peptides S4

Structures of the Complete Set of HAI Analogs S5

MALDI-TOF MS Spectra of the Pure HAI Analogs S7

^1^H-NMR Spectra of the Pure HAI Analogs S17

Peptide Stability in Human Serum S27

Parallel Artificial Membrane Permeability Assay (PAMPA) S28

RP-HPLC coupled to PDA and MALDI-TOF MS Quantification Results S29

Abbreviations S36

References S38

**Solid-Phase Peptide Synthesis**


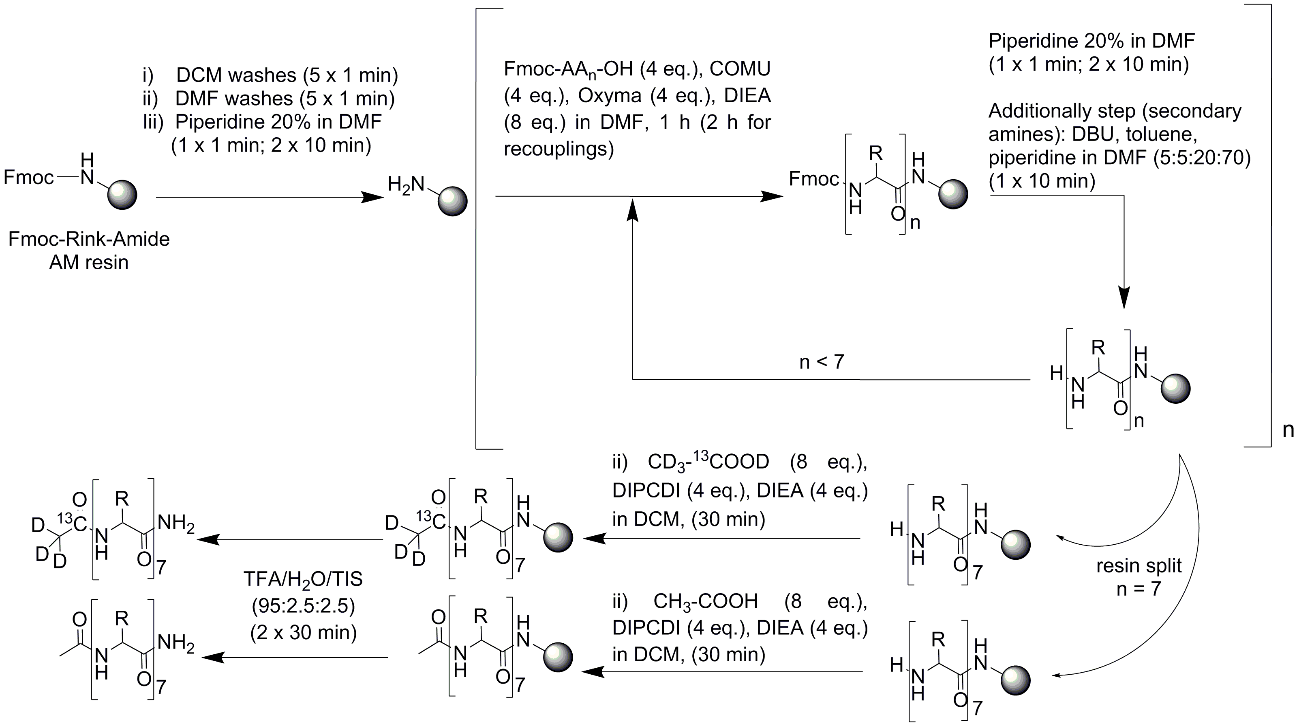


**Scheme S1.** Strategy for the synthesis of the complete set of HAI analogs with a final splitting step that allows differential isotopic labeling by means of acetylation.

**Characterization of the Peptides**

Total yields (*L* and *H*, respectively): **1L**, 11 and 15%; **2L**, 15 and 16%; **3L**, 12 and 8%; **4L**, 69 and 54%; **5L**, 55 and 48%; **6D**, 58 and 63%; **7-D**, 53 and 77%; **7+D**, 70 and 66%; **8D**, 10 and 5%; **9D**, 29 and 12%.

High resolution mass spectroscopy (LTQ-FT Ultra/Synapt HDMS; *L* and *H* versions, respectively): **1L**, HRMS calcd. for C_43_H_64_N_15_O_9_/ C_42_^13^C_1_H_61_^2^H_3_N_15_O_9_ [M+H]^+^, 934.5006/ 938.5228; found, 934.5010/ 938.5236; **2L**, HRMS calcd. for C_47_H_70_N_15_O_9_/ C_46_^13^C_1_H_67_^2^H_3_N_15_O_9_ [M+H]^+^, 988.5475/ 992.5697; found, 988.5482/ 992.5690; **3L**, HRMS calcd. for C_44_H_64_N_15_O_9_/ C_43_^13^C_1_H_61_^2^H_3_N_15_O_9_ [M+H]^+^, 946.5006/ 950.5227; found, 946.5027/ 950.5231; **4L**, HRMS calcd. for C_43_H_65_N_16_O_8_/ C_42_^13^C_1_H_62_^2^H_3_N_16_O_8_ [M+H]^+^, 933.5166/ 937.5388; found, 933.5200/ 937.5429; **5L**, HRMS calcd. for C_43_H_63_N_15_O_8_F_1_/ C_42_^13^C_1_H_60_^2^H_3_N_15_O_8_F_1_ [M+H]^+^, 936.4963/ 940.5184; found, 936.5001/ 940.5162; **6D**, HRMS calcd. for C_43_H_64_N_15_O_9_/ C_42_^13^C_1_H_61_^2^H_3_N_15_O_9_ [M+H]^+^, 934.5006/ 938.5228; found, 934.5046/ 938.5210; **7-D**, HRMS calcd. for C_49_H_68_N_15_O_9_/ C_48_^13^C_1_H_65_^2^H_3_N_15_O_9_ [M+H]^+^, 1010.5319/ 1014.5541; found, 1010.5290/ 1014.5558; **7+D**, HRMS calcd. for C_49_H_68_N_15_O_9_/ C_48_^13^C_1_H_65_^2^H_3_N_15_O_9_ [M+H]^+^, 1010.5319/ 1014.5541; found, 1010.5325/ 1014.5559; **8D**, HRMS calcd. for C_44_H_66_N_15_O_9_/ C_43_^13^C_1_H_63_^2^H_3_N_15_O_9_ [M+H]^+^, 948.5162/ 952.5384; found, 948.5162/ 952.5397; **9D**, HRMS calcd. for C_43_H_63_N_14_O_9_S_1_/ C_42_^13^C_1_H_60_^2^H_3_N_14_O_9_S_1_ [M+H]^+^, 951.4618/ 955.4840; found, 951.4603/ 955.4844.

| **Peptide ID**  **(*L* and *H*, respectively)** | **MW**  **(g/mol)** | **HPLC t_R_**  **(min)** | **Purity***^a^*  **(%)** | **MALDI-TOF**  **[M+H]^+^** |
| --- | --- | --- | --- | --- |
| **1L** | 933.5/ 937.5 | 3.60/ 3.69 | 97/ 97 | 934.4/ 938.4 |
| **2L** | 987.5/ 991.6 | 3.87/ 3.84 | 100/ 100 | 988.5/ 992.5 |
| **3L** | 945.5/ 949.5 | 3.72/ 3.72 | 100/ 100 | 946.4/ 950.4 |
| **4L** | 932.5/ 936.5 | 3.41/ 3.45 | 94/ 94 | 933.4/ 937.7 |
| **5L** | 935.5/ 939.5 | 4.03/ 4.00 | 95/ 96 | 936.6/ 940.6 |
| **6D** | 933.5/ 937.5 | 3.38/ 3.37 | 95/ 94 | 934.6/ 938.6 |
| **7+D** | 1009.5/ 1013.6 | 3.74/ 3.73 | 95/ 95 | 1010.6/ 1014.6 |
| **7-D** | 1009.5/ 1013.6 | 3.80/ 3.79 | 94/ 95 | 1010.6/ 1014.6 |
| **8D** | 947.5/ 951.5 | 3.47/ 3.46 | 99/ 99 | 948.6/ 952.6 |
| **9D** | 950.5/ 954.5 | 3.65/ 3.64 | 98/ 97 | 951.6/ 955.5 |

*^a^*After purification by RP-HPLC.

**Table S1.** Characterization of HAI analogs studied by RP-HPLC at 220 nm (gradient from 0 to 100% CH_3_CN in 8 min; SunFire C_18_ column) and MALDI-TOF.

**Structures of the Complete Set of HAI Analogs**

|   **1L** – Parent peptide |  |
| --- | --- |
|   **2L** – L-isoleucine substitution by homocyclohexyl-L-alanine |  |
|   **3L** – L-tyrosine substitution by 7-hydroxy-(S)-1.2.3.4-tetrahydroisoquinoline-3-carboxylic acid |  |
|   **4L** – L-tyrosine substitution by 4-amino-L-phenylalanine |  |
|   **5L** – L-tyrosine substitution by 4-fluoro-L-phenylalanine |  |
|   **6D** – *Retro*-D-version of the parent peptide |  |
|   **7+D** – D-proline substitution by (2S, 3S)-3-phenylpirrolidine-2-carboxylic acid |  |
|   **7-D** – D-proline substitution by (2R, 3R)-3-phenylpirrolidine-2-carboxylic acid |  |
|   **8D** – D-proline substitution by D-pipecolic acid |  |
|   **9D** – D-histidine substitution by 4-thiazoyl-D-alanine |  |

**Figure S1.** Structure of the complete set of HAI analogs.

**MALDI-TOF MS Spectra of the Pure HAI Analogs**

| ****  ***L*-1L** |
| --- |
| ****  ***H*-1L** |
| ****  ***L*-2L** |
| ***H*-2L** |
| ***L*-3L** |
| ***H*-3L** |
| ***L*-4L** |
| ***H*-4L** |
| ***L*-5L** |
| ***H*-5L** |
| ***L*-6D** |
| ***H*-6D** |
| ***L*-7+D** |
| ***H*-7+D** |
| ***L*-7-D** |
| ***H*-7-D** |
| ***L*-8D** |
| ***H*-8D** |
| ****  ***L*-9D** |
| ****  ***H*-9D** |

**Figure S2**. MALDI-TOF spectra of the pure HAI analogs (x axis units as [m/z]).

**^1^H-NMR Spectra of the Pure HAI Analogs**

| ****  **1L** |
| --- |
| ****  **2L** |
| ****  **3L** |
| ****  **4L** |
| ****  **5L** |
| ****  **6D** |
| ****  **7+D** |
| ****  **7-D** |
| ****  **8D** |
| ****  **9D** |

**Figure S3**. ^1^H-NMR spectra of the pure HAI analogs (x axis units as ppm). Peptides are paired by isotopic isomers (*i.e.* heavy and light peptides are displayed on the upper and the lower part of the spectrum, respectively).

**Peptide Stability in Human Serum**

**Figure S4**. Stability assay in human serum of peptides HAI (1L), *retro*-D-HAI (6L), 7-D and 8D. The parent peptide 1L, formed by L-amino acids, shows exponential degradation (R^2^ = 0.812, t_1/2_ = 3.3 h; non-linear regression ($Y=\left( Y_{0}-plateau \right)*e^{-KX}+plateau$); $Y_{0}$ and $plateau$ forced at 100 and 0, respectively), while D-peptides show stability to proteases (t_1/2_ > 24 h).

**Parallel Artificial Membrane Permeability Assay (PAMPA)**

Permeability is considered excellent with values >4.0 × 10^−6^ cm/s, uncertain between 2.0 × 10^−6^ and 4.0 × 10^−6^ cm/s, and poor with values below 2.0 × 10^−6^) cm/s^1^. Thus, as observed in Figure S5 and Table S2, all the peptides display a poor passive diffusion permeability.

|  |
| --- |
| **Figure S5.** – Passive diffusion of the peptides 1L, 6D, 7-D and 8D studied by PAMPA (n = 3). |

| **Peptide ID** | ***Pe* · 10^6^ (cm/s)** | **SD** |
| --- | --- | --- |
| **1L** | 0.18 | 0.11 |
| **6D** | 0.34 | 0.06 |
| **7-D** | 0.33 | 0.16 |
| **8D** | 0.34 | 0.08 |

**Table S2.** Passive diffusion (effective permeability) of the peptides 1, 6D, 7-D and 8D studied by PAMPA (n = 3).

**RP-HPLC coupled to PDA and MALDI-TOF MS Quantification Results**

| **Peptide ID** | **RP-HPLC-PDA** | | **MALDI-TOF MS** | |
| --- | --- | --- | --- | --- |
|  | **Transport (%)** | ***P_app_* (**× **10^6^ cm/s)** | **Transport (%)** | ***P_app_* (**× **10^6^ cm/s)** |
| **1L** | 2.9 ± 1.4 | 2.5 ± 1.1 | 3.8 ± 1.4 | 3.2 ± 1.2 |
| **2L** | 3.9 ± 0.9 | 3.3 ± 0.7 | 3.4 ± 0.7 | 2.8 ± 0.6 |
| **3L** | 2.0 ± 0.8 | 1.7 ± 0.6 | 2.5 ± 1.2 | 2.1 ± 1.0 |
| **4L** | 2.0 ± 0.9 | 1.6 ± 0.8 | 2.1 ± 0.5 | 1.8 ± 0.4 |
| **5L** | 3.4 ± 1.6 | 2.9 ± 1.3 | 3.1 ± 1.6 | 2.6 ± 1.3 |
| **6D** | 5.3 ± 1.8 | 4.5 ± 1.5 | 3.1 ± 0.9 | 2.6 ± 0.7 |
| **7+D** | 6.8 ± 1.2 | 5.7 ± 1.0 | 7 ± 2 | 5.7 ± 1.9 |
| **7-D** | 5.7 ± 1.8 | 4.8 ± 1.5 | 7 ± 3 | 6 ± 2 |
| **8D** | 7.2 ± 0.9 | 6.1 ± 0.8 | 7.5 ± 1.0 | 6.3 ± 0.8 |
| **9D** | 4.4 ± 0.5 | 3.7 ± 0.4 | 2.9 ± 1.2 | 2.4 ± 1.0 |

**Table S3**. Transport values (mean ± SD; n = 3) from the *in vitro* bovine BBB cell-based model assay, either quantified through RP-HPLC-PDA or by MALDI-TOF MS. Results are presented as transport (%) or apparent permeability (cm/s).

|  |
| --- |
| **Figure S6**. Transport (replicates) from the *in vitro* bovine BBB cell-based model assay, either quantified through RP-HPLC-PDA or by MALDI-TOF MS. Results are presented as transport (%). |
|  |

| **a**   |
| --- |
| **b**   |

**Figure S7**. Transport quantification for peptide 1L, second replicate: (**a**) RP-HPLC-PDA quantification at 220 nm (gradient from 0 to 100% CH_3_CN in 8 min; SunFire C_18_ column) fell within the limit of detection (LOD) although not within the limit of quantification (LOQ), and (**b**) MALDI-TOF MS quantification of this sample is within the LOQ.

|  |
| --- |

**Figure S8**. Refinement of RP-HPLC-PDA detection of the peptide HAI (1L – not acetylated). Absorption spectra of an injection at diverse wavelengths: 200, 210 and 220 nm (gradient from 0 to 100% CH_3_CN in 8 min; SunFire C_18_ column).

| **Wavelength (nm)** | **Signal-to-noise ratio**  **normalized by the lowest value** |
| --- | --- |
| 200 | 1.00 |
| 210 | 1.18 |
| 220 | 1.26 |

**Table S4**. Signal-to-noise ratio determined for the peptide HAI (1L – not acetylated) at diverse wavelengths: 200, 210 and 220 nm. Data obtained from the chromatogram shown in **Figure S12**. The signal-to-noise ratio is higher at 220 nm than at the other two wavelengths.

| **a** |
| --- |
| **** |
| **b** |
| **** |
| **c** |
| **** |
| **d** |
| **** |

**Figure S9**. Transport quantification spectra for peptides (**a**, **b**) 1L and (**c**, **d**) 2L. It can be observed that spectra of (**a**, **c**) time zero samples is cleaner than (**b**, **d**) acceptor wells (second replicate of 1L and first replicate of 2L, respectively).

**Abbreviations**

^1^H-NMR proton nuclear magnetic resonance

ACH *α*-cyano-4-hydroxycinnamic acid

BBB blood-brain barrier

BPLE brain polar lipid extract

CNS central nervous system

COMU 1-cyano-2-ethoxy-2-oxoethylidenaminooxy)dimethylamino-morpholino-carbenium hexafluorophosphate

CPP cell-penetrating peptide

DBU 1,8-diazabicyclo[5.4.0]undec-7-ene

DCM dichloromethane

DIEA *N,N*-diisopropylethylamine

DIPCDI *N,N'*-diisopropylcarbodiimide

DMEM Dulbecco's modified Eagle medium

DMF dimethylformamide

ECM endothelial cell medium

ESI electrospray ionization mass spectrometry

Fmoc 9-fluorenylmethoxycarbonyl

*H* heavy version of the peptide

HBSS Hanks’ balanced salt solution

HEPES 4-(2-hydroxyethyl)-1-piperazineethanesulfonic acid

HRMS high-resolution mass spectrometry

*L* light version of the peptide

LOD limit of detection

LOQ limit of quantification

LY lucifer yellow lithium salt

MALDI-TOF MS matrix-assisted laser desorption/ionization time-of-flight mass spectrometry

MS mass spectrometry

MTBE methyl *tert*-butyl ether

NMR nuclear magnetic resonance

Oxyma Pure ethyl (hydroxyimino)cyanoacetate

PAMPA parallel artificial membrane permeability assay

*P_app_* apparent permeability

*P_e_* effective permeability

PDA photodiode array

Pro proline

*retro*-D-version peptide made of D-amino acids and with the inverted sequence of a parent peptide

RP-HPLC reversed-phase high-performance liquid chromatography

SD standard deviation

SPPS solid-phase peptide synthesis

*T* transport

*t*Bu *tert*-butyl

TEER transendothelial electrical resistance

TFA trifluoroacetic acid

Tf transferrin

TfR transferrin receptor

TIS triisopropylsilane

t_R_ retention time

UV/Vis ultraviolet/visible

**References**

1 Di, L., Kerns, E. H., Fan, K., McConnell, O. J. & Carter, G. T. High throughput artificial membrane permeability assay for blood–brain barrier. *Eur. J. Med. Chem.* **38**, 223-232, (2003).
